# Whole‐exome sequencing predicted cancer epitope trees of 23 early cervical cancers in Chinese women

**DOI:** 10.1002/cam4.953

**Published:** 2016-12-20

**Authors:** Xia Li, Hailiang Huang, Yanfang Guan, Yuhua Gong, Cheng‐Yi He, Xin Yi, Ming Qi, Zhi‐Ying Chen

**Affiliations:** ^1^ The Laboratory for Gene and Cell EngineeringShenzhen Institutes of Advanced TechnologyChinese Academy of SciencesShenzhenChina; ^2^ Research Center for Biomedical Information TechnologyShenzhen Institutes of Advanced TechnologyChinese Academy of SciencesShenzhenChina; ^3^ Analytic and Translational Genetics UnitMassachusetts General HospitalBostonMassachusetts; ^4^ Broad Institute of MIT and HarvardCambridgeMassachusetts; ^5^ Geneplus‐BeijingBeijing102206China; ^6^ BGI‐ShenzhenShenzhenChina; ^7^ School of Basic Medical SciencesCenter for Genetic and Genomic MedicineZhejiang University Medical School 1st Affiliated Hospital James Watson Institute of Genome SciencesHangzhouZhejiangChina; ^8^ Department of Pathology and Laboratory MedicineUniversity of Rochester Medical CenterRochesterNew York

**Keywords:** Cervical cancer, immunotherapeutic target, neo‐epitope, phylogenetic tree

## Abstract

Emerging evidence suggest that the heterogeneity of cancer limits the efficacy of immunotherapy. To search for optimal therapeutic targets for enhancing the efficacy, we used whole‐exome sequencing data of 23 early cervical tumors from Chinese women to investigate the hierarchical structure of the somatic mutations and the neo‐epitopes. The putative neo‐epitopes were predicted based on the mutant peptides’ strong binding with major histocompatibility complex class I molecules. We found that each tumor carried an average of 117 mutations and 61 putative neo‐epitopes. Each patient displayed a unique phylogenetic tree in which almost all subclones harbored neo‐epitopes, highlighting the importance of individual neo‐epitope tree in determination of immunotherapeutic targets. The alterations in *FBXW7* and *PIK3CA*, or other members of the significantly altered ubiquitin‐mediated proteolysis and extracellular matrix receptor interaction related pathways, were proposed as the earliest changes triggering the malignant progression. The neo‐epitopes involved in these pathways, and located at the top of the hierarchy tree, might become the optimal candidates for therapeutic targets, possessing the potential to mediate T‐cell killing of the descendant cells. These findings expanded our understanding in early stage of cervical carcinogenesis and offered an important approach to assist optimizing the immunotherapeutic target selection.

## Introduction

Immunotherapy is emerging as the most promising type of cancer treatment, as evidenced by recent clinical trials in which durable remission, and even cure, have been demonstrated in some patients. However, its success is limited because only a small proportion of patients respond to the therapies, whereas most remain resistant and are either unresponsive or responsive only transiently 1, 2. A growing body of evidence suggests that cancer heterogeneity is the bottleneck that limits the efficacy of immunotherapy 3. Although derived from a single initiated cell, almost all cancers comprise multiple subclones and evolve constantly, driven by mechanisms such as genome instability and Darwinian selection. Some recent cancer genome studies have revealed that subclones of a cancer comprise different compositions of genomic alterations 4, 5. It is conceivable that some of these alterations have become the determinants of whether the subclone responds to or resists current immunotherapies, including immune checkpoint inhibitors, tumor infiltration lymphocytes, chimeric antigen receptor‐modified T cells, and bispecific antibodies. Each of these immunotherapy strategies targets only one or a few subpopulations and allows the others, especially the metastatic ones, to thrive continuously 6. It is also conceivable that these genetic variabilities have become the bottleneck that limits clinical efficacy; many patients either have no response or have an incomplete response in which the cancer shrinks or even disappears but eventually recurs despite continuing treatment.

It has become apparent that the development of technology to target all of a tumor's subclones is necessary to remove the bottleneck and bring cancer immunotherapy to a new level. We recently proposed the construction of a “cancer epitope tree” to achieve this goal 3 because, although the cancer genome is highly variable both spatially and temporally, the technology is available to determine the subclonal hierarchical structure, the so‐called phylogenetic tree, to outline the temporal relationship among the genomic alterations 7, even at the single‐cell level 8. In combination with the technique to predict the neo‐epitopes created by these genomic alterations, a “cancer neo‐epitope tree” can be constructed to guide the systematic search for the optimal therapeutic targets located at the “trunk” or “major branch” that possess the potential to mediate T‐cell killing of all or most of the cancer cells 3. With few exceptions 9, most cancer phylogenetic trees were constructed with driver mutations at a cohort level. Passenger mutations outnumber driver mutations by up to 2000 times 4, 10, 11; many of them are target candidates and could play an important role in causing cancer cell death by cytotoxic T lymphocytes and antibody‐dependent cell‐mediated cytotoxicity. Therefore, for purposes of immunotherapy, it is important to include neo‐mutated epitopes derived from both driver and passenger mutations. Furthermore, an accumulating body of evidence suggests that each cancer is unique in its composition of genetic alterations and hierarchical structure of subclones 7, 9. Therefore, a “cancer epitope tree” of an individual cancer will be more useful than the one at cohort level for determination of immunotherapeutic targets.

In this article, we report the results from the first attempt to construct the individual “cancer epitope tree” of 23 early cervical cancers in Chinese women by exploring their exome sequencing data. Cervical cancer is the most lethal cancer in women worldwide, with an estimated 528,000 new cases and 266,000 deaths in 2012 12. Persistent infection with high‐risk human papillomavirus (HPV) subtypes, such as HPV 16 and 18, has been found in a majority of patients with cervical cancer. Although multiple studies have characterized the mutation landscape of cervical cancer 13, 14, the molecular events responsible for malignant transformation remain elusive. Because the 23 cervical cancers in this study were at very early stages (17 stage I ([T1, N0, M0]: the cancer has grown into (invaded) the cervix, but it is not growing outside the uterus. The cancer has not spread to nearby lymph nodes (N0) or distant sites (M0)) and 6 stage II ([T2, N0, M0]: the cancer has grown beyond the cervix and uterus, but has not spread to the walls of the pelvis or the lower part of the vagina), according to the International Federation of Gynecology and Obstetrics [FIGO] staging system 15), they allowed us to study the early events in cervical carcinogenesis while defining the optimal immunotherapeutic targets. We found that the samples from 73.9% of our patients with early‐stage cervical cancer carried a few integrated HPV sequences. The ubiquitin proteolysis and extracellular matrix (ECM) receptor pathways were significantly altered; 69.6% of cancers carried alterations in these two pathways. We propose that alterations in genes *FBXW7* and *PIK3CA* are more likely to serve as the early genomic mutations that cause the progression of HPV‐induced precancerous cells toward invasive malignancy. Furthermore, we used the identified somatic mutations to predict the neo‐epitopes on the basis of their mutated peptides’ binding affinities with major histocompatibility complex class I molecules (MHC‐I). Using this information, we constructed the phylogenetic tree and the “cancer epitope tree” for individual tumors. We found that the mutations of individual tumors displayed a unique path of evolution, highlighting its importance in the search for therapeutic targets. HPV proteins might serve as immunotherapy targets in tumors that carry the integrated virus genome without active HPV infection. However, for tumors that do not express these proteins, our approach will suggest desirable therapeutic target candidates. The results of this study expanded our understanding of the early stages of cervical carcinogenesis and, more importantly, offered a useful systematic strategy with which to search for the optimal immunotherapeutic targets, which has important implications for cancer diagnosis, prevention, and therapy.

## Materials and Methods

### Sample collection and preparation

Twenty‐three pairs of cervical cancer tumors and matched normal tissues were obtained from the Southwest Hospital of Chongqing Autonomous Municipality in China. The study protocol was approved by the Institutional Review Board of Southwest Hospital, and all experiments were performed in accordance with the guidelines and regulations. Informed consent was obtained from each subject. Tumors and peripheral blood samples were collected from patients S1‐S20, who each underwent surgical resection. For patients S21, S22, and S23, adjacent tissues were used as the control samples. The surgically resected tumors were snap frozen in liquid nitrogen and stored at −80°C. The blood samples were stored at −20°C. DNA was extracted from the frozen tissues and peripheral blood lymphocytes using commercial kits (TIANamp Blood DNA Kits and Genomic DNA Kits, Tiangen Biotech) and following the manufacturer′s instructions. HPV genotyping was performed using the polymerase chain reaction (PCR)‐based mass spectrometry system 16.

### Whole‐exome sequencing

DNA from matched tumor and control samples were fragmented with an ultrasonicator UCD‐200 (Diagenode). These fragments were purified and size selected with Ampure Beads (Beckman, Beverly, MA) following three enzymatic steps (end repairing, the addition of an “A” base, and adapter ligation) according to Illumina's instructions. NimbleGen EZ 64M human exome array probes (SeqCap EZ Human Exome Library v3.0) were used in hybridization. Each captured library was then pair‐end sequenced in 100‐bp lengths with an Illumina HiSeq 2000 following the manufacturer's instructions. The raw data are available from the corresponding authors upon reasonable request and with permission of Beijing Genomics Institute.

### Read mapping and somatic mutation detection

Raw whole‐exome sequencing reads were aligned to the reference human genome (hg19) using a BWA aligner (v 0.7.10) 17 with default parameters. Alignments were sorted and converted into BAM format. Picard (v1.119) (http://picard.sourceforge.net/) was used to mark possible PCR duplicates in the BAM file, and the Genome Analysis Toolkit (v3.2.2) 18 was used to improve alignment accuracy. Somatic point mutations were detected with MuTect (v1.1.4) 19. Variants from the 1000 Genome Project (Phase 3) 20, the NHLBI GO Exome Sequencing Project (version 2) 21, which represented variants from more than 200,000 individuals, and the Exome Aggregation Consortium (version 0.2) 22, which spanned variants from 60,706 unrelated individuals (with a minor allele frequency threshold of 0.1), were removed from the somatic mutations. Variants were annotated for effects on transcripts using the variant effector predictor tool 23.

### Validation of somatic mutations

We validated a subset of recurrent mutations together with some randomly selected mutations by either mass spectrum or Sanger sequencing. Specific primers were designed for PCR amplification and base extension that covered the mutation sites. Genotyping assay and base calling procedures were performed on the MassArray platform of Sequenom by determining their genotypes in the tumors and matched samples. The PCR amplification products were sequenced with a 3730xl DNA Analyzer (Applied Biosystems). All sequences were analyzed with Sequencing Analysis Software Version 5.2 (Applied Biosystems, Foster City, CA).

### HPV genome alignment

The reads that could not be mapped to the human reference genome were extracted and realigned to a database of multiple HPV reference genomes. HPV reference genomes were obtained from the Human Papilloma Virus Episteme (pave.niaid.nih.gov) 24. With the paired‐end read information, we determined whether the HPV genome could integrate into the human genome by screening pairs of reads with one end mapped to the human genome and the other end mapped to the HPV genome.

### Pathway analysis

The KEGG pathways were obtained from the Molecular Signatures Database (MSigDB) 25, and the gene set was downloaded from http://www.broadinstitute.org/gsea/downloads.jsp (accessed 19 June 2015). The mutated genes in each tumor were compared with the KEGG pathway to determine whether the tumor had altered pathways.

For each pathway, we randomly sampled the same number of genes from all genes in the human genome without replacement. We then counted the number of tumors that harbored mutation in this random gene set. We performed 10,000 such random samplings for each pathway and calculated the *P*‐value as the proportion of random samples in which more patients carried mutations than the number of tumors that used the original pathway. The false discovery rate was then calculated for each pathway using the Benjamini and Hochberg method. The significantly enriched pathway was considered if the adjusted *P* < 0.1.

### Phylogenetic inference

The evolutionary history of each of the 23 tumors was constructed on the basis of the somatic mutations’ reads count using PhyloSub 26. This approach made use of Bayesian inference and Markov chain Monte Carlo sampling (with 2500 samplings) to estimate the number of clonal lineages and their ancestry. We only considered trees with the highest likelihood.

We downloaded 194 cervical cancers’ mutation data and the RNA‐sequencing data from the International Cancer Genome Consortium (ICGC) data portal (https://dcc.icgc.org). For these mutations, we did one same filtration using the variants from the 1000 Genome Project, the NHLBI GO Exome Sequencing Project, and the Exome Aggregation Consortium. We then merged all the mutations into one matrix of genes versus tumor samples with 0/1 entries indicating the absence/presence status of a mutation in a gene for each sample. Based on this matrix, we used the BML 27 tool to infer the sequence of gene mutations for the 194 cervical cancer data.

### Immunogenic variants prediction

For each somatic missense mutation, we obtained the corresponding mutated amino acid and one peptide centred on the mutated residue, flanked on each side by eight amino acids from the protein sequence. We also obtained the corresponding normal 17 amino acid peptide. We then used the NETMHC‐3.4 algorithm 28 to predict the binding affinity for the peptide with MHC‐I. The variant showed immunogenicity only if the mutated peptide showed strong binding affinity with MHC‐I (affinity < 50) and the normal peptide had no binding affinity (affinity > 500) at the same peptide position.

### Gene expression analysis

The normalized read counts from the ICGC donors’ RNA‐sequencing data from the 194 cervical cancer tissues, with the log transformation, were calculated as the gene expression values. Gene set enrichment analysis (GSEA) 25, 29 using the KEGG pathway, the canonical pathway, and the biological process gene set was performed separately to identify pathways that have significantly altered expression levels in carriers of the *FBXW7* or *PIK3CA* mutants. The significantly enriched pathway was considered if the *q* < 0.05.

The difference in *FBXW7* and *PIK3CA* was tested using two‐sided Student's *t* test. Pearson correlation was used to assess the correlation between *FBXW7* and *PIK3CA* gene expression. All analyses were performed with the R Version 3.1.1 statistical software (R Core Team, Vienna, Austria). A two‐sided *P *< 0.05 was considered to indicate statistical significance.

## Results

### General data

Our study included specimens of 23 patients who received surgical treatment upon diagnosis of early‐stage cervical cancer (17 stage I and 6 stage II, according to the FIGO staging system; Table 1) in the Southwest Hospital of Chongqing Autonomous Municipality in China. We performed whole‐exome sequencing of 242,232 exons, with a length of 63.8 megabases, at an average coverage of 181X (Fig. S1). Peripheral blood samples from most patients were used as germline controls, with the exception of patients S21, S22, and S23, for whom adjacent tissues were used instead. We detected HPV sequences in all but two tumor samples and found only a small number of integrated sequences in 17 exome‐captured sequencing datasets (Table S1). We used MuTect and Indelocator 19, 30 to call each case's somatic mutations by filtering out germline events from the corresponding normal sample. We also filtered out variants in the 1000 Genome Project (Phase 3) 20, the NHLBI GO Exome Sequencing Project (version 2) 21, and the Exome Aggregation Consortium (version 0.2) 22 by applying a minor allele frequency threshold of 0.1 to all three databases. The final cleaned dataset includes 2691 somatic mutations, including 730 synonymous substitutions, 1934 nonsynonymous substitutions, 18 deletions, and 9 insertions across 23 sample pairs. A subset of 59 somatic mutations was selected for validation, and 57 variants (96.6%) were validated using mass spectrum or Sanger sequencing (Table S2). The number of nonsynonymous mutations showed no correlation with the patients’ age or clinical stage (Fig. S2).

**Table 1:** Clinical stage and human papillomavirus (HPV) infection status of 23 patients with cervical cancer

Tumor sample code	Age (years)	Clinical stage	HPV genotyping
S1	54	Ib1	HPV16
S2	46	Ib1	HPV16
S3	44	Ib2	HPV16
S4	49	Ib1	HPV16
S5	43	Ib1	HPV16
S6	39	Ib1	HPV16
S7	42	Ib1	HPV16
S8	38	Ib1	HPV33
S9	48	Ib1	HPV16
S10	50	IIa1	HPV16
S11	46	Ib1	HPV16
S12	44	Ib1	HPV16
S13	41	Ib1	HPV16
S14	56	Ib1	HPV18
S15	48	Ib1	HPV16
S16	44	Ib1	HPV18
S17	44	IIa1	HPV18
S18	37	Ib1	HPV16
S19	35	Ib1	HPV16
S20	49	IIa1	HPV16
S21	59	IIa1	HPV16
S22	46	IIb	HPV16
S23	63	IIa1	HPV16

### Frequency of mutations in cervical cancer

**Figure 1:**
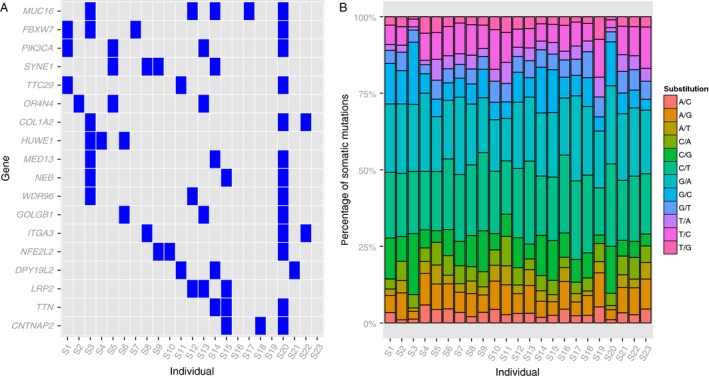
Distribution of mutated genes and base substitution patterns in the 23 patients with cervical cancer. (A) Distribution of mutated genes in at least three patients (mutation frequency > 13%). Each column represents one individual, and each row is a gene. (B) Distribution of base substitution patterns for all somatic mutations.

We first estimated the distribution of the somatic mutations and their nucleotide substitutions. C/T and G/A substitutions were the most frequent among the 23 patients (Fig. 1B), with mean frequencies of 21.8% and 21%, respectively. This observation, especially the C/T substitution pattern, agrees with findings from a previous study of 115 Norwegian and Mexican cervical cancer samples 13. We found that *PIK3CA* (17.4%), *SYNE1* (17.4%), *FBXW7* (17.4%), and *MUC16* (21.7%) were among the most frequently mutated genes (Fig. 1A), which again agrees with the findings of previous studies: the Norwegian and Mexican study 13 showed that *EP300* (16%), *FBXW7* (15%), and *PIK3CA*(14%) harbored recurrent mutations; a study 31 in 80 cervical cancer samples from Boston showed that *PIK3CA* (31.3%), *KRAS* (8.8%), and *EGFR* (3.8%) had the highest mutation rates; and another study in 15 cervical cancer patients from Hong Kong revealed frequent alteration of *FAT1* (33.3%), *ARID1A* (33.3%), *ERBB2* (26.7%), and *PIK3CA* (53.3%) 32. We obtained 194 cervical cancer whole‐exome sequencing mutation datasets from ICGC. After applying the same filtering, we also noticed *PIK3CA*(27.3%) and *FBXW7*(10.3%) (Fig. S3). Despite the ethnic and geographic differences, alterations in *PIK3CA*, followed by *FBXW7*, were the most common mutations in the various cervical cancer studies.

### Prediction of neo‐epitopes

**Figure 2:**
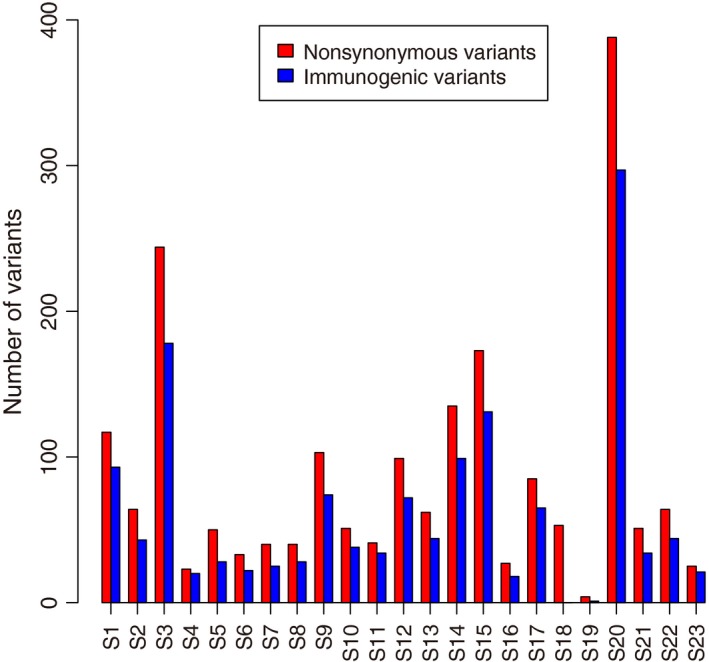
Number of immunogenic variants in 23 patients. The numbers of total nonsynonymous mutations (red) and immunogenic variants (blue) are shown for each patient. The immunogenic variants were predicted only if the mutated peptide showed strong binding affinity with MHC‐I (affinity < 50) and the normal peptide had no binding affinity (affinity > 500) at the same peptide position.

The mutated peptides derived from those mutations, if presented on MHC‐I, could potentially be immunogenic as the adaptive immune system should recognize them as “non‐self” neo‐antigens. Recent neo‐antigen prediction approach by estimating the binding affinity between the mutated peptide and MHC‐I can be used to identify therapeutic targets for immunotherapy 33, 34, 35, 36. Using the same approach (see Methods), of the 1934 nonsynonymous substitutions, we found 1405 missense mutations’ peptides exhibiting strong binding affinity with MHC‐I (Fig. 2), which suggests that tumor progression could generate antigens that may recruit immunologic cells to attack the tumor cells.

### Alteration in ubiquitin‐mediated proteolysis and ECM receptor interaction pathways

To determine whether any biological functions were significantly altered, we integrated all of the nonsynonymous mutations from the 23 patients and determined the pathways that were enriched. In permutation tests of 10,000 samples among the 177 mutated pathways, the ubiquitin‐mediated proteolysis and ECM receptor interaction pathways were the most significantly altered, with false discovery rates of <0.1 (Table 2). All of the mutated genes involved in these two pathways are shown in Figure S4.

**Table 2:** Top 10 altered pathways

KEGG Pathway	Number of patients altered	*P* value	FDR
UBIQUITIN‐MEDIATED_PROTEOLYSIS	15	0.0014	0.072
ECM_RECEPTOR_INTERACTION	11	0.0016	0.072
INSULIN_SIGNALING_PATHWAY	14	0.0028	0.1008
HYPERTROPHIC_CARDIOMYOPATHY_HCM	9	0.0036	0.108
FOCAL_ADHESION	14	0.0044	0.1131
DILATED_CARDIOMYOPATHY	9	0.0065	0.14625
ALDOSTERONE_REGULATED_SODIUM_REABSORPTION	7	0.0075	0.15
MTOR_SIGNALING_PATHWAY	8	0.0124	0.2094
SMALL‐CELL_LUNG_CANCER	10	0.0128	0.2094
INOSITOL_PHOSPHATE_METABOLISM	9	0.0146	0.219

The ubiquitin‐mediated proteolysis pathway mediates protein degradation via the ubiquitin conjugation and proteasome system. It is reported to be the most frequently altered pathway in clear cell renal cell carcinomas and a contributor to the tumorigenesis 37. In our cervical cancer data, the mutated genes involved in this pathway included *FBXW7* (altered in 17%), *HUWE1* (altered in 13%), and *BIRC6* (altered in 9%). It has been suggested that mutations in *FBXW7* cause increased genetic instability because several prominent oncogenes (*Notch*,* c‐Myc*,* JunB*, and *mTOR*) are its substrates 38, 39. In cervical cancer, the ubiquitin‐mediated proteolysis pathway can be best characterized by high‐risk HPV‐16 E6 binding activity to the tumor‐suppressor protein p53 to induce ubiquitylation and proteasomal degradation 40, 41, and the abrogation of p53 allows the accumulation of genetic mutations that would normally have been repaired. The HPV‐18 E7 oncoprotein also targets the tumor‐suppressor *Rb* proteins for proteasomal degradation via the ubiquitin‐dependent pathway 42. Although the mTOR signaling pathway is not the most significant, it is among the top 10 altered pathways (with *PIK3CA* altered in 17% of patients). Thus, the alterations in genes involved in the ubiquitin‐mediated proteolysis pathway may trigger a cascade of reactions that lead to malignancy.

We found that, in the ECM receptor interaction pathway, genetic alterations mainly occurred in *COL1A2* (altered in 13%) and *ITGA3* (altered in 13%). These alterations may disrupt the signaling transfer function during interactions with extracellular proteins, leading to malfunction in cellular activities such as adhesion, migration, differentiation, proliferation, and apoptosis. This pathway was also involved in the focal adhesion pathway comprising mutations in *PIK3CA* (altered in 17%), *COL1A2* (13%), and *ITGA3* (13%). HPV‐positive cells have been found to express high levels of focal adhesion kinase, which regulates the interaction between the signal transduction of ECM and integrins 43. The virus oncoprotein HPV‐16 E6 also binds to the ECM protein, leading to cytoskeletal reorganization and formation of focal adhesions 44. This interaction, in combination with deregulation of focal adhesion kinase, promotes resistance to anoikis and allows the HPV‐infected cells to proliferate in the absence of adherence to the ECM, that is, anchorage‐independent growth 45. Thus, the altered genes in these pathways may allow cells to escape anoikis and play a role in transformation and tumor invasion.

### The cancer neo‐epitope trees

**Figure 3:**
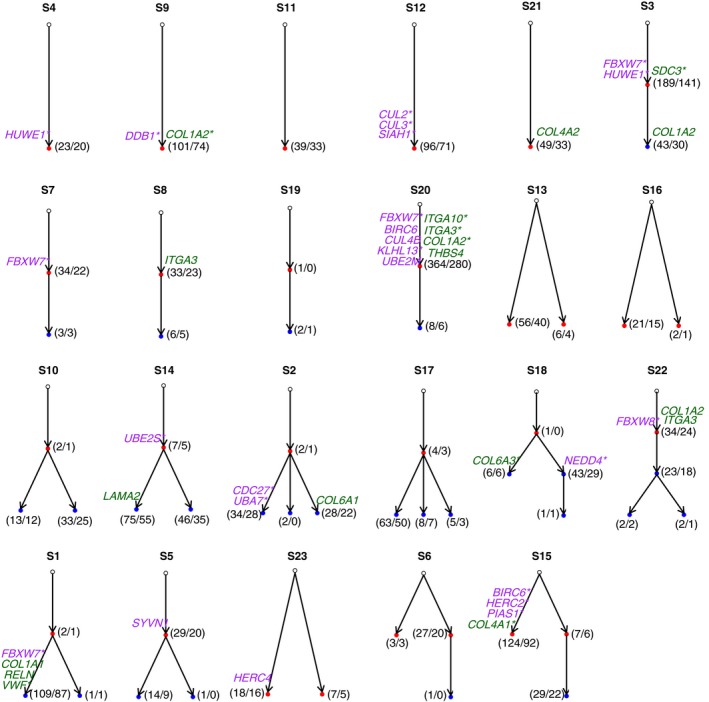
The clonal structures and phylogenic relationships for all 23 patients. In each patient, a phylogenic tree was constructed using somatic mutations. Each node represents one clone. Each clone harbors multiple mutations, and only the genes involved in the ubiquitin‐mediated proteolysis (purple) and ECM receptor interaction (dark green) pathways are labeled on the corresponding node. Normal clones (non‐pathogenic) are in open circles, early clones are shown in red, and later clones are shown in blue. Arrows point from the parent node to the child node (i.e., the descendant clone derived from the ancestor clone). Asterisks indicate that the gene harbors putative neo‐epitopes. The numbers in parentheses indicate the number of total mutated genes (before the slash) and the number of genes that harbored putative neo‐epitopes in the clone.

Tumors usually contain multiple genetically diverse clones or subclones that have constantly evolved from an earlier population through expansion and selection 4, 46, 47. Outlining the evolutionary history of these mutations will aid in understanding the cancer development and guide design of therapy targets 48. We therefore constructed phylogenetic trees for each tumor using nonsynonymous substitutions (Fig. 3, Table S3) and named the clones in chronical order as the ancestor, descendant, and later subclones. Consistent with their early stage of malignancy, the subclonal hierarchy structures of all tumors were simple. Five tumors (S4, S9, S11, S12, and S21) harbored only one ancestor subclone, and no descendants were observed. The evolutionary paths in the other tumors showed either linear (S3, S7, S8, S19, and S20) or branching (the remaining 13) patterns. Five of the 13 tumors with branching paths (S6, S13, S15, S16, S23) had two ancestor subclones (S6 and S15 derived one descendant subclone from one of the two ancestors), and the other eight carried only one ancestor subclone with multiple descendants or later subclones (Fig. 3).

Each tumor displayed accumulation of different mutations and evolutionary paths over time, suggesting heterogeneity between patients. Thus, for therapeutic considerations, the individual phylogenetic tree should offer clues for the selection of therapy targets. The number of altered genes in each subclone and the number of genes that harbored neo‐epitopes are shown in Figure 3. Each tumor carried an average of 117 mutations and 67 antigenic targets. All tumors but S19, which only harbored three mutations, had subclones that harbored neo‐epitopes, which makes immunotherapy a feasible approach. An individualized “cancer epitope tree” could be constructed using neo‐epitopes. Selection of targets in the ancestor subclones would inhibit the majority of the tumor cells because the descendants are derived from the ancestor subclone. We defined the ancestor subclones as the “trunk” and the descendant subclones as the “major branches” in the phylogenic tree. Among the many neo‐epitopes in the trunk and major branches, one possibility to choose functional mutations or to scale down the mutation is to choose the genes involved in important pathways. We therefore list in Figure 3 the 34 altered genes involved in the ubiquitin‐mediated proteolysis and ECM receptor interaction pathways in these trees, together with the number of neo‐epitopes.

### Alteration of *FBXW7* and *PIK3CA*

**Figure 4:**
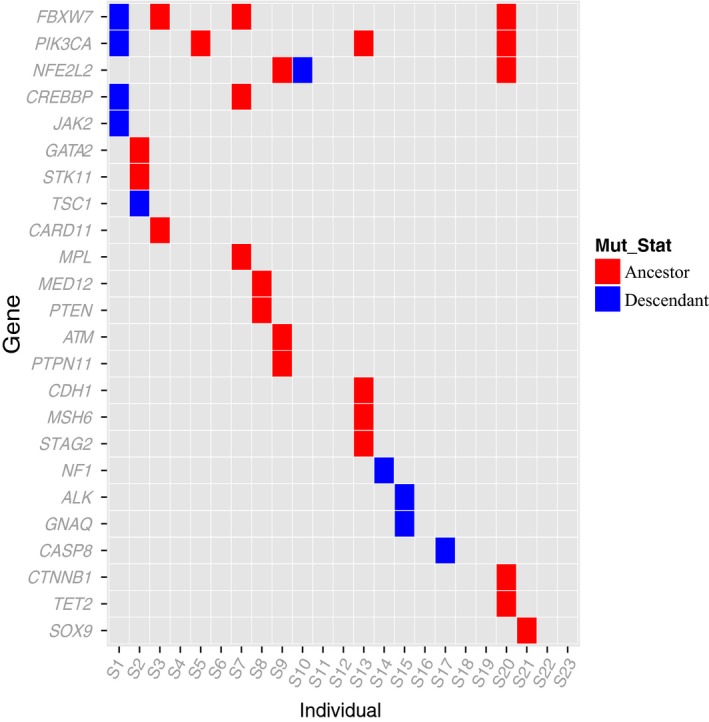
Distribution of the reported driver genes in our 23 patients. Driver genes were obtained from the literature 49. The ancestral (red) or descendant (blue) status for each gene mutation in each patient was labeled.

Mutations of both passenger and driver genes occur during a lesion's transition from precancerous to malignant. Among the approximately 20,000 protein‐coding genes in the human genome, only 138 genes were reported in a previous study as driver genes 49, which play a significant role in tumorigenesis. We found that 24 of the 138 proposed driver genes were mutated in our 23 tumors (Fig. 4). In addition to *FBXW7* (17%) and *PIK3CA* (17%), which were the most frequent, *NFE2L2* (13%) and *CREBBP* (9%) were also frequently mutated. *NFE2L2* participates in protein processing and amino acid metabolism and was recently identified in a recent cervical cancer study 13. Interestingly, in tumor S2, two driver genes, *GATA2* and *STK11*, were located in the ancestor subclone, and *STK11* also harbors neo‐epitope (Table S3). It is possible that the two ancestral mutated driver genes granted a selective growth advantage to allow the cancer cells to derive more descendants in S2, as we observed. In the S2‐specific “epitope tree,” *STK11*, which is part of the mTOR signaling pathway, may be a “trunk” target candidate.

Overall, *FBXW7* and *PIK3CA* seem to play more important roles in these early‐stage tumors. Both showed mutations in four patients. In the individual phylogenetic trees, both genes were located on the ancestor subclones in three tumors, which suggests they were likely early events during tumorigenesis. Due to the limited number of samples available in our study, we made one extension study on the mutations of 194 ICGC cervical cancer donors which also represented the cervical cancer's pathogenesis. We inferred the likely sequence of mutation for these patients. The results supported the symptom that *FBXW7* and *PIK3CA* mutations were more likely to be the second mutation event starting at the normal status, with probability 0.86 and 0.97 separately (Fig. S5). We propose that alterations in *FBXW7* and *PIK3CA* are likely the early changes that trigger the progression of the HPV‐induced precancerous cells toward invasive malignancy.

Using the genome‐wide gene expression from ICGC donors’ RNA‐sequencing data, we performed GSEA between the mutant and wild‐type for these two genes. No pathway or biological process was found to significantly differ in carriers of the mutants (Tables S4, S5). We further compared the expression levels of *FBXW7* and *PIK3CA* between carriers of the mutants and the wild‐types. We found the mutated *FBXW7*′s expression was significantly increased (Student's *t* test, *P*: 0.006231; Fig. S6A) while *PIK3CA* generally showed no changes (Student's *t* test, *P*: 0.1293; Fig. S6B). When comparing *PIK3CA* expression in *FBXW7* mutant carriers and wild‐type, we found *FBXW7* mutants were marginally associated with *PIK3CA* expression levels (Student's *t* test, *P*: 0.02241; Fig. S6C), and *FBXW7*′s expression displayed no changes in *PIK3CA* mutant and wild‐type cases (Student's *t* test, *P*: 0.4807; Fig. S6D). The expression of these two genes was significantly correlated (Fig. S7). Further investigations with a larger sample size and in relevant tissue and cell lines are needed to reach a convincing conclusion.

## Discussion

Cervical cancer is among the few malignancies that allows convenient study from morphologic, cytological, and molecular events during the formation of precancerous lesions and their transition to invasive cancers. Consequently, timely diagnosis and treatment of early‐stage cervical cancer is possible. The whole‐exome sequencing data in this study were obtained from 23 patients with early‐stage cervical cancer (FIGO stage I or II) to allow identification of early genomic events without the complication of late‐stage genomic alterations. Infection of high‐risk HPV is a prerequisite for cervical cancer, and integration of the viral genome occurs throughout the course of carcinogenesis. Twenty of the 23 patients had infection with high‐risk HPV, and integrated HPV sequences were detected in 17 cases, albeit at relatively low sequences covered. A total of 2691 genomic alterations, mostly single nucleotide substitutions, were identified, of which 1405 were predicted to encode neo‐epitopes on the basis of their strong binding affinity with MHC‐I. Each cancer carried an average of 117 nonsynonymous somatic mutations and 61 predictive neo‐epitopes. To outline the phylogenetic relationship among these somatic mutations, we constructed the subclonal hierarchical structures of individual tumors and named the identified cancer cell populations in temporal order as ancestor, descendant, and late subclones. We found that five patients carried only the ancestor subclone, 16 carried an additional descendant, and only two had all three subclones. Furthermore, we found that 17% of the tumors had mutations in *PIK3CA* and *FBXW7* without mutation of typical driver genes, such as *KRAS*,* TP53,* and *EGFR,* as reported in other cervical cancer genome studies 13, 31; these were not found in our study, which suggests that they may be the later‐stage events.

It has been well documented that HPV viral oncoproteins E6 and E7 can induce precancerous lesions and that additional genetic alterations are required for malignant transformation 50. Through our exome sequencing data, although we observed multiple HPV‐related sequences, we detected only a small number of HPV integrated sequences, while in some patients there was no observation. Currently, we do not have enough evidence that either whether the whole HPV genome is integrated with the host cell or a part of them is integrated by the exome capture technique. The low HPV integrated sequences may confirm that most of the cervical cancers in this study were at a very early stage of malignancy. Therefore, the somatic mutations in our samples provide more information that they must have been capable of triggering the transition from benign to invasive lesions. We here highlighted the early somatic mutations for critical targets selection. Based on our analyses, we propose that mutation of *FBXW7* and *PIK3CA* and other members in these two pathways were among the earliest alterations that triggered malignant transformation. This hypothesis is consistent with earlier studies in a large number of cancer types in which mutation of *FBXW7* and *PIK3CA* was a frequent event, including cancers of the colon, brain, gastrointestinal system 51, 52, cervix 32, head and neck 53, and breast 54. This hypothesis is further supported by recent evidence that mutated *PIK3CA* initiates breast cancer by triggering multiple key events during the cancer initiation stage 55.

Our findings will be very useful in guiding cancer immunotherapy. A growing body of evidence suggests that cancer heterogeneity is the bottleneck that limits the efficacy of cancer immunotherapy 6, 56. Most of the current immunotherapeutic technologies, including tumor infiltration lymphocytes, immune checkpoint inhibitors, chimeric antigen receptor‐modified T cells, and bispecific antibodies, kill only one or a few subclones in a cancer and allow the others to continue to grow. In our study, we found that each of the 23 cervical tumors had a unique subclonal hierarchical structure that comprised a different composition of genetic alterations and predicted neo‐epitopes. Therefore, the “cancer neo‐epitope tree” of each tumor is critical to help determine the optimal targets at the trunk or major branch shared by all descendant cells that have the potential to lead to a cure.

Another important observation is that a large number of passenger mutations encoded neo‐epitopes that were potential target candidates. Many earlier studies also demonstrated that each cancer encodes a unique set of genetic alterations, but they focused on driver mutations and demonstrated the phylogenic tree at the cohort level without indicating their temporal relationship; thus, they are very useful in outlining cancer signal pathways, but not in determining the most suitable therapeutic target. Passenger mutations greatly outnumber driver mutations, so that they may play an important role in cancer immunotherapy 57, 58. Conceivably, the “cancer neo‐epitope tree” strategy as established in this study will help to determine optimal therapeutic targets and result in a great increase in clinical efficacy or even cure, especially when a cocktail of targets is used to reduce the chances of escape due to sporadic loss of the targets.

In this study, we constructed the “cancer neo‐epitope tree” using genomic data derived from a single DNA sample from each tumor. It should be noted that this approach is limited by many factors, such as the heterogeneous composition of the tumor's cell population, the exome capture efficiency, the genomic sequencing and assembly technique, and the tumor cell collection method. Therefore, our technique is able to draw out a “cancer neo‐epitope tree” that comprises only the major subpopulations. Even so, integrity is achievable only when the tumor is small and DNA is fully representative. For a large tumor, however, DNA from well‐designed multiple samples 4, 11 would be more appropriate. Currently, we predict the immunogenic mutations based on their mutated peptides’ binding affinity with the MHC‐I. We should note that further immunogenic experiments are warranted to validate the real immunogenicity of those putative neo‐epitopes so that we can better test our idea.

In summary, our results show that each tumor carried a unique set of genetic alterations and associated putative epitopes and that the construction of individual “cancer epitope trees,” together with the earliest genomic events, such as alterations in *FBXW7*,* PIK3CA,* and other members in the pathways, could assist in the understanding of the early genetic events involved in cervical carcinogenesis and, more importantly, the systematic search for optimal immunotherapeutic targets at the trunk or major branches.

## Conflict of Interest

The authors declare no competing financial interests.

## Supporting information


**Figure S1.** Distribution of alignment rate and coverage. Bar plot shows the alignment rate (A) and coverage (B) in each tumor (red) and control (blue) sample for each patient.

**Figure S2**. Correlation of mutations with patients’ age and stage. (A) Distribution of patients’ nonsynonymous mutations number with the age. The correlation coefficient and the significance *P*‐value are shown. (B) Distribution of patients’ nonsynonymous mutations number with the disease stage of the patients. *P* value was calculated by two‐sided Student's t test (mean ± S.D.; *n* = 23 subjects).

**Figure S3.** Distribution of mutated genes among 194 donors from ICGC. Distribution of mutated genes in 194 cervical cancer patients (mutation frequency > 8.2%). Each column represents one individual, and each row is a gene.

**Figure S4.** Mutated genes involved in the two significantly altered pathways. The heat map shows the mutated genes in the 23 patients involved in the two significantly altered pathways: the ubiquitin‐mediated proteolysis (red) and ECM receptor interaction pathways (blue).

**Figure S5.** The temporal order of mutations in the 194 ICGC cervical cancer donors. Beginning with the normal circle, figure shows all possible sequence of somatic mutated genes after leaving out low‐probability (the probability from the parent circle to the child circle below 0.4) events. The arrow points to possible order among mutated genes. Color for each circle is scaled according to the relative probability from the parent circle to the child circle (the color scale probability labeled under each panel).

**Figure S6.** Relationship between *FBXW7* and *PIK3CA* mRNA expression and mutation statuses. Distribution of the gene expression values of *FBXW7* (A) and *PIK3CA* (B) in patients harboring mutant and the wild‐type, separately. *PIK3CA* or *FBXW7* mRNA expression levels were also compared between samples with *FBXW7* (C) or *PIK3CA* (D) mutant and wild‐type forms. The normalized read counts from the ICGC donors’ RNA‐sequencing data, with the log transformation, were calculated as the gene expression values. *P* value was calculated by two‐sided Student's *t* test (mean ± S.D.; *n* = 194 subjects). Mut, mutant; Wld, wild‐type.

**Figure S7.** Significant correlation between *FBXW7* and *PIK3CA* expression.

Table S1. Number of reads mapped to the HPV genomes.

Table S2. Validated mutations.

Table S3. Somatic mutations with the phylogenic relationship, immunogenicity, and altered pathways.

Table S4A. KEGG pathway enrichment of GSEA report for mutant *FBXW7*.

Table S5A. KEGG pathway enrichment of GSEA report for mutant *PIK3CA*.
