# Supplementary material for: Whole‐exome sequencing predicted cancer epitope trees of 23 early cervical cancers in Chinese women
Source: Cancer Med. 2016 Dec 20;6(1):207–19. doi: 10.1002/cam4.953 (PMC5269563; doi:10.1002/cam4.953)
Supplement: Supplementary file 8 — Table S1. Number of reads mapped to the HPV genomes. [file CAM4-6-207-s008.doc]

Table S1. Number of reads mapped to the HPV genomes

| Sample | Total HPV reads | HPV type | HPV integration reads (pair) |
| --- | --- | --- | --- |
| S1 | 2126 | HPV16 | 6 |
| S2 | 22 | HPV16 | 11 |
| S3 | 2 | HPV16 | 1 |
| S4 | 3 | HPV16 | 3 |
| S5 | 2960 | HPV16 | 9 |
| S6 | 9 | HPV16 | 0 |
| S7 | 1 | HPV16 | 1 |
| S8 | 1416 | HPV33 | 2 |
| S9 | 207 | HPV16 | 1 |
| S10 | 1945 | HPV16 | 11 |
| S11 | 5399 | HPV16 | 13 |
| S12 | 1778 | HPV16 | 9 |
| S13 | 91 | HPV16 | 0 |
| S14 | 4912 | HPV18 | 14 |
| S15 | 105 | HPV16 | 1 |
| S16 | 0 | / | 0 |
| S17 | 6 | HPV18 | 0 |
| S18 | 25 | HPV16 | 1 |
| S19 | 0 | / | 0 |
| S20 | 122 | HPV16 | 0 |
| S21 | 810 | HPV16 | 3 |
| S22 | 1831 | HPV16 | 16 |
| S23 | 112 | HPV16 | 2 |
