# Supplementary material for: Whole‐exome sequencing predicted cancer epitope trees of 23 early cervical cancers in Chinese women
Source: Cancer Med. 2016 Dec 20;6(1):207–19. doi: 10.1002/cam4.953 (PMC5269563; doi:10.1002/cam4.953)
Supplement: Supplementary file 9 — Table S2. Validated mutations. [file CAM4-6-207-s009.doc]

Table S2. Validated mutations

| Patient | Gene | GenomePosition | CodonChange | AminoAcidChange | Validation | Method |
| --- | --- | --- | --- | --- | --- | --- |
| S1 | *FBXW7* | chr4:153249385 | Cgt/Tgt | R465C | somatic | mass spectrum |
| S1 | *PIK3CA* | chr3:178936091 | Gag/Aag | E545K | somatic | mass spectrum |
| S1 | *CDON* | chr11:125893307 | tCt/tTt | S22F | somatic | mass spectrum |
| S1 | *CREBBP* | chr16:3824629 | Cgt/Tgt | R742C | somatic | mass spectrum |
| S1 | *TET1* | chr10:70406602 | gaA/gaC | E1372D | somatic | Sanger |
| S2 | *AHNAK* | chr11:62296386 | Gat/Aat | D1835N | somatic | Sanger |
| S2 | *STK11* | chr19:1226486 | gGa/gCa | G381A | somatic | mass spectrum |
| S2 | *LRP1B* | chr2:141571375 | gGt/gTt | G1737V | somatic | Sanger |
| S3 | *FBXW7* | chr4:153247289 | Cgc/Ggc | R505G | somatic | mass spectrum |
| S3 | *COL1A2* | chr7:94049591 | gGa/gAa | G709E | somatic | Sanger |
| S3 | *HUWE1* | chrX:53674349 | Gag/Cag | E105Q | somatic | mass spectrum |
| S3 | *MED13* | chr17:60088043 | aAt/aGt | N612S | somatic | mass spectrum |
| S3 | *NEB* | chr2:152411543 | Gaa/Caa | E6444Q | somatic | mass spectrum |
| S3 | *KIAA1549* | chr7:138522707 | Gag/Aag | E1933K | somatic | mass spectrum |
| S3 | *NIN* | chr14:51273464 | Caa/Gaa | Q86E | somatic | mass spectrum |
| S3 | *PAPPA2* | chr1:176526157 | aaG/aaC | K233N | somatic | mass spectrum |
| S3 | *RYR1* | chr19:39019297 | Gac/Cac | D3666H | somatic | mass spectrum |
| S3 | *STAT3* | chr17:40486030 | Caa/Gaa | Q279E | somatic | mass spectrum |
| S4 | *HUWE1* | chrX:53570870 | Gag/Aag | E3771K | somatic | mass spectrum |
| S4 | *OBSCN* | chr1:228511109 | Cag/Tag | Q6109* | somatic | mass spectrum |
| S5 | *PIK3CA* | chr3:178936082 | Gaa/Aaa | E542K | somatic | mass spectrum |
| S5 | *SYNE1* | chr6:152647507 | Cag/Aag | Q5073K | somatic | mass spectrum |
| S5 | *CEP290* | chr12:88513893 | gTg/gGg | V507G | somatic | mass spectrum |
| S5 | *FAT2* | chr5:150892037 | cGc/cAc | R3865H | No mutation | Sanger |
| S6 | *NUP214* | chr9:134019942 | Ccc/Tcc | P524S | somatic | mass spectrum |
| S8 | *SYNE1* | chr6:152774783 | Ctt/Ttt | L989F | somatic | mass spectrum |
| S8 | *MED12* | chrX:70348270 | Gat/Tat | D1112Y | somatic | mass spectrum |
| S8 | *PTEN* | chr10:89692904 | Cga/Tga | R130* | somatic | Sanger |
| S9 | *CDK12* | chr17:37665977 | Gat/Cat | D877H | somatic | mass spectrum |
| S9 | *KMT2D* | chr12:49425638 | Cag/Tag | Q4284* | somatic | Sanger |
| S9 | *PTPN11* | chr12:112926308 | Gag/Cag | E481Q | somatic | mass spectrum |
| S10 | *NFE2L2* | chr2:178098810 | Gag/Cag | E79Q | somatic | mass spectrum |
| S10 | *HYDIN* | chr16:71186645 | gGa/gTa | G253V | somatic | mass spectrum |
| S10 | *MUC19* | chr12:40836951 | aCg/aTg | T745M | somatic | Sanger |
| S12 | *WDR96* | chr10:105945768 | aaA/aaT | K658N | somatic | Sanger |
| S12 | *AHNAK2* | chr14:105416693 | Ggg/Tgg | G1699W | somatic | Sanger |
| S13 | *PIK3CA* | chr3:178936091 | Gag/Aag | E545K | somatic | mass spectrum |
| S13 | *CEP290* | chr12:88512453 | caG/caC | Q530H | somatic | mass spectrum |
| S13 | *OBSCN* | chr1:228509651 | Gag/Aag | E5994K | somatic | mass spectrum |
| S13 | *TSC2* | chr16:2126522 | Gat/Cat | D925H | somatic | mass spectrum |
| S14 | *SYNE1* | chr6:152651115 | aGa/aCa | R4902T | somatic | mass spectrum |
| S14 | *BUB1B* | chr15:40501898 | Gag/Cag | E736Q | somatic | mass spectrum |
| S14 | *NF1* | chr17:29685515 | tCt/tTt | S2663F | somatic | mass spectrum |
| S14 | *MED13* | chr17:60042559 | tCa/tTa | S1551L | somatic | Sanger |
| S14 | *CGNL1* | chr15:57810672 | Gag/Aag | E898K | somatic | Sanger |
| S14 | *VPS13D* | chr1:12460307 | Gag/Cag | E3902Q | somatic | Sanger |
| S15 | *NEB* | chr2:152534095 | aCg/aTg | T1253M | somatic | mass spectrum |
| S15 | *CELSR1* | chr22:46763726 | cTg/cCg | L2660P | somatic | Sanger |
| S15 | *COL4A1* | chr13:110835427 | Gga/Aga | G670R | somatic | Sanger |
| S15 | *IGFN1* | chr1:201180820 | Gat/Cat | D2267H | somatic | mass spectrum |
| S18 | *ANK2* | chr 4:114290858 | aGa/aAa | R3836K | somatic | Sanger |
| S20 | *FBXW7* | chr4:153249384 | cGt/cAt | R465H | somatic | mass spectrum |
| S20 | *COL1A2* | chr7:94041951 | gGa/gTa | G487V | somatic | Sanger |
| S20 | *MED13* | chr17:60088194 | Caa/Gaa | Q562E | somatic | Sanger |
| S20 | *MED13* | chr17:60088317 | Cag/Gag | Q521E | somatic | Sanger |
| S20 | *NLRC5* | chr16:57062247 | atC/atG | I731M | somatic | mass spectrum |
| S20 | *WDR96* | chr10:105990394 | atC/atG | I91M | No mutation | Sanger |
| S21 | *CSMD2* | chr1:33999410 | cCa/cTa | P3182L | somatic | Sanger |
| S22 | *COL1A2* | chr7:94039756 | Cct/Act | P372T | somatic | Sanger |
