# Supplementary material for: Whole‐exome sequencing predicted cancer epitope trees of 23 early cervical cancers in Chinese women
Source: Cancer Med. 2016 Dec 20;6(1):207–19. doi: 10.1002/cam4.953 (PMC5269563; doi:10.1002/cam4.953)
Supplement: Supplementary file 13 [file CAM4-6-207-s013.doc]

**Supplementary figure legend:**

Figure S1. Distribution of alignment rate and coverage.

Barplot shows the alignment rate (A) and coverage (B) in each tumor (red) and control (blue) sample for each patient.

Figure S2. Correlation of mutations with patients’ age and stage.

(A) Distribution of patients’ nonsynonymous mutations number with the age. The correlation coefficient and the significance p-value are shown. (B) Distribution of patients’ nonsynonymous mutations number with the disease stage of the patients. P value was calculated by two-sided Student’s t test (mean ± s.d.; n = 23 subjects).

Figure S3. Distribution of mutated genes among 194 donors from ICGC.

Distribution of mutated genes in 194 cervical cancer patients (mutation frequency > 8.2%). Each column represents one individual, and each row is a gene.

Figure S4. Mutated genes involved in the two significantly altered pathways.

The heatmap shows the mutated genes in the 23 patients involved in the two significantly altered pathways: the ubiquitin mediated proteolysis (red) and ECM receptor interaction pathways (blue).

Figure S5. The temporal order of mutations in the 194 ICGC cervical cancer donors.

Beginning with the normal circle, figure shows all possible sequence of somatic mutated genes after leaving out low-probability (the probability from the parent circle to the child circle below 0.4) events. The arrow pointing means possible order among mutated genes. Color for each circle is scaled according to the relative probability from the parent circle to the child circle (the color scale probability labeled under each panel).

Figure S6. Relationship between *FBXW7* and *PIK3CA* mRNA expression and mutation statuses.

Distribution of the gene expression values of *FBXW7* (A) and *PIK3CA* (B) in patients harboring mutant and the wild type, separately. *PIK3CA* or *FBXW7* mRNA expression levels were also compared between samples with *FBXW7* (C) or *PIK3CA* (D) mutant and wild-type forms. The normalized read counts from the ICGC donors’ RNA-sequencing data, with the log transformation, were calculated as the gene expression values. P value was calculated by two-sided Student’s t test (mean ± s.d.; n = 194 subjects). Mut, mutant; Wld, wild-type.

Figure S7. Significant correlation between *FBXW7* and *PIK3CA* expression.
